# Investigation of Selected Surface Topography Parameters and Deformation during Milling of Vertical Thin-Walled Structures from Titanium Alloy Ti6Al4V

**DOI:** 10.3390/ma16083182

**Published:** 2023-04-18

**Authors:** Szymon Kurpiel, Krzysztof Zagórski, Jacek Cieślik, Krzysztof Skrzypkowski

**Affiliations:** 1 Faculty of Mechanical Engineering and Robotics, AGH University of Science and Technology, Mickiewicza 30 Av., 30-059 Krakow, Poland; 2 Faculty of Civil Engineering and Resource Management, AGH University of Science and Technology, Mickiewicza 30 Av., 30-059 Krakow, Poland

**Keywords:** surface topography, surface deformation, vertical thin-walled structures, titanium alloy, milling, aerospace industry

## Abstract

Thin-walled elements are widely used in the aerospace industry, where the aim is to reduce the process time and the weight of the structure while ensuring the sufficient quality of the finished product. Quality is determined by geometric structure parameters and dimensional and shape accuracy. The main problem encountered during the milling of thin-walled elements is the deformation of the product. Despite the various methods available for measuring deformation, more are still being developed. This paper presents selected surface topography parameters and deformation of vertical thin-walled elements during an experiment under controlled cutting conditions for samples from titanium alloy Ti6Al4V. Constant parameters of feed (f), cutting speed (V_c,_) and tool diameter (D) were used. Samples were milled using a tool for general-purpose and a tool for high-performance machining, as well as two different machining approaches: with greater involvement of face milling, and cylindrical milling with a constant material removal rate (MRR). For samples with vertical thin walls, the parameters of waviness (W_a_, W_z,_) and roughness (R_a_, R_z_) were measured using a contact profilometer in the selected areas on both processed sides. Deformations were determined in selected cross-sections perpendicular and parallel to the bottom of the sample using GOM measurement (GOM—Global Optical Measurement). The experiment showed the possibility of measuring deformations and deflection arrows of thin-walled elements proceeded from titanium alloy using GOM measurement. Differences in selected surface topography parameters and deformations were observed for the machining methods used with an increased cross-section of the cut layer. A sample with a deviation of 0.08 mm from the assumed shape was obtained.

## 1. Introduction

The growing demand for transport, including air transport, forces manufacturers to increase the number of aircraft structures and to use structures with greater reliability and reduced costs. It is, therefore, necessary to improve and find new solutions for designing and manufacturing aircraft structures [1]. Changes in the aerospace industry are occurring rapidly, so the materials used and the processing technologies for these materials are changing rapidly. The reliability and durability of components used in the aerospace industry largely depend on the type of material used [2]. Developments in this sector of the industry have resulted in the use of mainly titanium alloys and nickel alloys for stressed components of aerospace structures [3,4]. Despite using titanium and nickel alloys, other alternative aerospace materials are constantly being pursued in order to achieve increasingly favorable parameters of finished products while meeting strict requirements and ensuring appropriate mechanical properties [5]. One way to reduce costs is to use increasingly lighter materials with good properties, which will have a beneficial effect on weight reduction. Currently, there is a trend to use metal nanocomposites as a lightweight material that provides good properties, so that the weight of the finished product can be reduced [6]. In the aerospace industry, there is a strong development of hybrid or composite-metal materials in aerospace structures. The data show that composite materials continue to compete with metal materials in this industry [7].

The most commonly used types of non-ferrous metals in the aerospace industry are aluminum and aluminum alloys, magnesium and magnesium alloys, titanium and titanium alloys, copper and copper alloys, and nickel and nickel alloys [1,4,7]. Based on the use of titanium and nickel alloys, another way of reducing production costs is by reducing the weight of the structure while ensuring sufficient rigidity during the loads that can occur [8,9]. It is a reason that thin-walled structures are increasingly being used in the aerospace industry.

In the case of plates, is assumed the relationship between the length of the shorter side (p) and the wall thickness (h), is defined by the following relations [10]:

- Super-thin walls: h/p < 1/100;
- Thin walls: 1/100 ≤ h/p ≤ 1/5;
- Thick walls: h/p > 1/5.

Thin-walled structures are used in the aerospace industry in elements such as stringers, ribs, frames, spars, hubs, blisks, turbine blades, shells, bulkheads or skin panels. Thin-walled components are manufactured from materials used in the aerospace industry such as aluminum alloys, titanium alloys, and nickel alloys. Their mechanical properties ensure sufficient requirements while reducing weight. Another advantage of integrated thin-walled components is the reduction in fuel consumption, the reduction in the number of connections, the minimization of labor, and the reduction in assembly costs [11,12]. The interest in thin-walled machining is the subject of analysis by researchers. The data provide important information for the industry [13,14].

**Figure 1:**
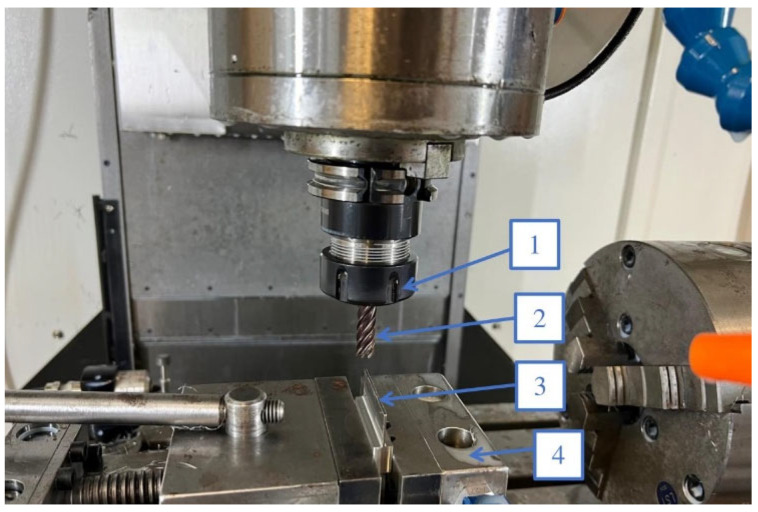
Experimental setup for milling vertical thin-walled samples: 1—tool holder, 2—tool, 3—workpiece, 4—vice.

One of the problems encountered during the processing of thin-walled elements is vibrations and forces, which as a result affect the quality of the finished product, including its surface roughness and dimensions [15,16,17,18]. Another problem that exists during the milling of thin-walled elements is the dimensional error resulting from the deflection of the object due to low rigidity. This problem does not occur when machining rigid parts and is related to the flexibility of the cutting system [11,19]. Shape deformations are a serious problem, as aerospace manufacturers strive to improve the quality of their products to remain competitive. This implies the use of increasingly narrow dimensional tolerances [16,20,21]. A diagram of a vertical thin-wall deformation during milling is shown in Figure 1.

The authors of the paper [22] analyzed the deformation of thin-walled elements with vertical walls of aluminum alloy EN AW7075 T651 using conventional milling and high-speed machining for finishing. The result of their studies was the measurement of deformation carried out using a coordinate measuring machine. Based on the presented data, the effect of the method on the obtained dimensional and shape accuracy is observed. It is also important to observe the variable value of deformations on the cross-section of the sample.

Authors in the work [23] tested the possibility of using a high-speed camera to control deformation during machining. Fixed cutting parameters were adopted and time displacements were tested for a fixed sample point. Tests were carried out for vertical thin-walled samples from aluminum alloy 7075 according to the parameters recommended by the tool manufacturer. The authors observed the effectiveness of using the camera in controlling the deformation of thin-walled elements, thanks to which they showed the possibility of using alternative measuring instruments in the diagnostics of product parameters. The paper also presents the appearance deviations of the thin wall occurring as a result of the machining of the element.

The authors of the paper [24] presented the possibility of using an optical method to measure the deformation of thin-walled elements. In their development, they made an aircraft element from aluminum alloy 7075 and measured the deformation using a GOM machine.

The machining of titanium alloy components is much more difficult than aluminum alloys due to the high cutting resistance, which is the result of high strength, high chemical reactivity, and low thermal conductivity [25,26].

The work by Gang [19] presented a deformation of thin-walled elements from titanium alloy on the vertical cross-section, for only one side of the machining surface. It was shown that a maximum part deflection equaled circa 0.1 mm in the middle of the wall.

In a study [27], thin-walled titanium alloy Ti6Al4V samples were milled using various support methods. During the experiment, cylindrical face milling was used during one-sided milling. The unsupported thin-wall machined sample was made with the following cutting parameters V_c_ = 40 m/min, a_p_ = 16 mm, a_e_ = 0.2 mm, f_z_ = 0.06 mm/rev for a tool of D = 12 mm. Based on the measurement, a maximum deviation of the thin wall of about 0.1 mm at a maximum height of 30 mm was noticeable.

Yusop et al. [28] presented samples of a curved vertical thin-walled element made using a trochoidal milling strategy. The workpiece material was titanium alloy Ti6Al4V. When machining with a 10 mm diameter cutter, they assumed a cutting speed of 47 m/min and 50 m/min (depending on the case), a feed per tooth of 0.03 mm/rev, and a trochoidal milling stepover of 1.6 mm. The result of their study was the presentation of the dimensional deviation of the sample at half-height at the selected point on both sides. The maximum deviation presented was 0.18 mm.

When machining parts, it is extremely important to choose the right cutting conditions. One way is to use information systems that provide access to information on machining conditions. High-quality companies use such systems that give the necessary information [29,30]. The term “information system” can be defined as a system of formalized procedures that enable the management of internal and external information, which is used for planning, management, and control purposes [19,22,23,24,25,26,27,28,29,30,31,32,33,34,35].

In the article [36], it was shown that the lack of an information system is the result of time losses in the aspect of cutting tool flow management. The lack of an information system resulted in frequent consultations with the rest of the team, which could have been eliminated by creating an appropriate database. Such time losses also increase costs. In the aspect of machining aerospace components, saving time is very important, since, as mentioned, cost reduction is emphasized in this industry [11,12].

The main goal of the current work was to test the applicability of the optical method GOM (Global Optical Measurement) measurement to determine the deformation of thin-walled samples. So far, this method has been used to measure the frame of an aluminum alloy aircraft, and the results were presented in the article [24]. The deflections will be determined over the entire height of the specimen in three selected sections (parallel and perpendicular to the bottom) on both sides of the machined area. The GOM method is an optical method, and therefore its application depends on the material and its dimensions being tested. Due to the use of thin-walled structures, care must be taken during the measurement to make an accurate measurement of the entire surface. An additional objective was to check the selected parameters of a thin-walled sample during machining with an increased section of the cut layer for possible use in finishing operations. During the experiment, two values of cutting depth and radial depth were used, using a constant material removal rate, cutting speed and feed rate. In the research, the titanium alloy Ti6Al4V was used, which is a popular material in the aircraft industry. The work benefited from the information system and knowledge of Seco Tools.

## 2. Materials and Methods

The sample series was prepared using a Mikron VCE 600 Pro milling center with iTNC 530 software. The experiment was carried out with two monolith milling tools with a diameter of ⌀10 supplied by Seco Tools. The first tool, JSE514100D2C.0Z4-SIRA, is dedicated to general application for machining all materials, while the second tool, JHP770100E2R040.0Z4A-SIRA, is dedicated to high-performance machining of titanium and nickel alloys. Table 1 presents the basic indicators of the used tools.

The JSE514100D2C.0Z4-SIRA monolithic face mill is a tool from the JS514 series with four cutting blades for which the lead angle is 0°. The geometry is characterized by two center cutting capability blades with uniform pitch, for which the chamfer at the corner is 0.1 × 45°. The flute helix angle for this tool is 35°. The cutter is mounted in the tool holder using a cylindrical shank type with a diameter of 10 mm using tolerance class h5. The tool does not contain an internal cooling channel, but for machining titanium alloys it is recommended to use coolant (emulsion), which was supplied by the machine. The geometry of the JSE514, based on the universal coating of the working part with SIRON-A, allows the machining of most materials for specialized applications, including titanium alloy [37]. JHP770100E2R040.0Z4A-SIRA is a four-blade monolithic tool from the JHP770 series designed for high-material-removal-rate machining. The tool features a defined groove shape and an uneven pitch with no center cutting capability blades. The blades contain a lead of 0° and a corner radius of 0.4 mm. The flute helix angle is 42°. The tool is mounted using a cylindrical shank type with a diameter of 10 mm. The tool is designed with a neck angle equal to 0° between the shank and the item for cutting, with a neck diameter of 9.4 mm and a neck length of 30 mm. In this geometry, it is possible to use an internal cooling channel, but during the experiment, coolant was supplied from the outside. Based on its geometry and coating with SIRON-A, the JHP770 series cutter is transparent for machining only titanium alloys [38]. The tools used had similar geometries, and the differences due to the design of the tool affected the type of materials used and the type of machining carried out [37,38]. Tool 1, which is the lower price, is designed for universal machining, while tool 2, which is almost three times the price, is intended only for special applications for machining titanium alloys. With this comparison, it can be decided which tool to choose for the presented machining to achieve the desired parameters.

The experiment was conducted under controlled cutting conditions with constant parameters: feed f = 255 mm/min and cutting speed V_c_ = 100 m/min. The cutting parameters adopted during the experiment were selected following the recommendations of the manufacturer of the tools [37,38]. During sample processing, the tool was mounted in the precision collet ⌀10 and placed in the tool holder ER32.

The samples were milled using water–oil emulsion SILUB MAX, which is a two-component coolant product that meets the requirements of TRGS 611. During the experiment, a mixture of 15% oil emulsion and 85% water was used, according to the manufacturer’s recommendations. The coolant is designed for universal applications, including the processing of special materials under extreme conditions [39]. The experimental setup for milling the vertical thin-walled samples is shown in Figure 1.

The object of the studies was thin-walled elements with vertical walls made of titanium alloy Ti6Al4V. This material is widely used in various industries. One of the most popular applications is use in aircraft structure [40]. The chemical composition and the mechanical properties of Ti6Al4V are shown in Table 2 and Table 3.

**Figure 2:**
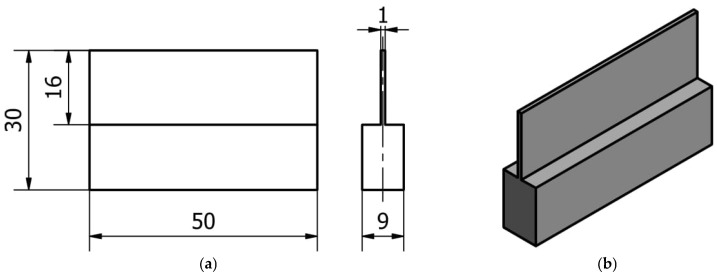
Documentation of vertical thin-walled sample: (**a**) design documentation; (**b**) 3D model in isometric view.

The blank for the sample preparation was a sheet of dimensions 9 mm × 30 mm × 50 mm. The thin wall with a dimension of 1 mm was made with a length of 50 mm and a height of 16 mm, according to the documentation shown in Figure 2. For consistent MMR during processing, the sample was pre-ground to a dimension of 9 mm. The sample was mounted in a vice at a height of 10 mm and supported from underneath by ground metal sheets.

Two different side milling approaches were used for the preparation of the samples: with more involvement of face milling, and with more involvement of cylindrical milling. In the first approach, a larger radial depth (a_e_ = 4 mm) was used for greater engagement of the tool face and cutting depth a_p_ = 2 mm. In the second approach, a larger cutting depth (a_p_ = 16 mm) was used for greater engagement of the cylindrical tool part and a radial depth a_e_ = 0.5 mm. For both cases, a constant material removal rate was adopted and equaled MRR = 2.03 cm^3^⁄min. To determine the material removal rate, the following relationship was used (1), which included depth of cut a_p_ (mm), radial depth a_e_ (mm), and feed rate V_f_ (mm/rev) [41]. The tools and depths of each case are shown in Table 4.

MRR = a_p_ · a_e_ · V_f_ (1)

**Figure 3:**
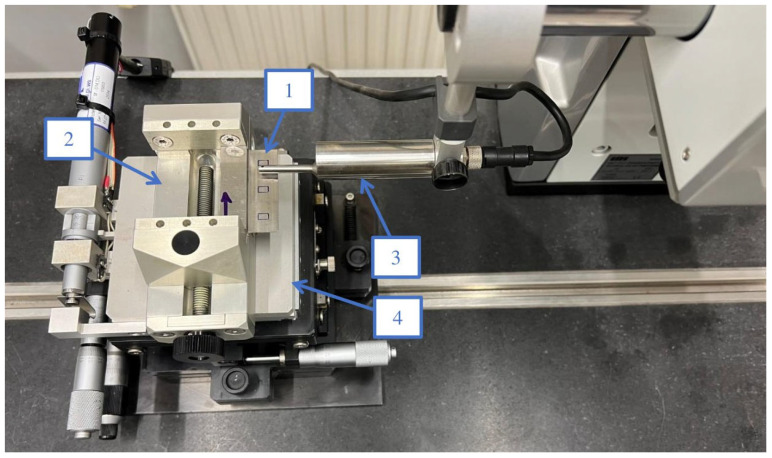
The method of measuring surface topography vertical thin-wall samples: 1—sample, 2—vice, 3—stylus tip, 4—translation stage.

In the first step, the basic parameters of the geometric structure of the surface were measured for the prepared samples. Surface parameters were determined by the contact method using the contact profilometer Topo 01P v3D. The method of measuring surface topography vertical thin-wall samples is presented in Figure 3.

**Figure 4:**
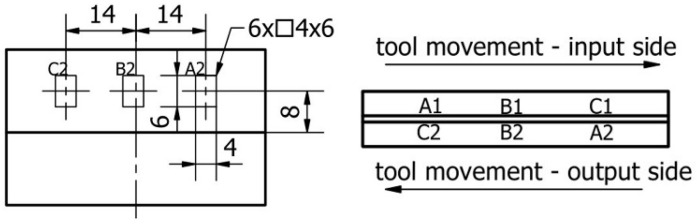
The marking of the measuring areas during surface topography measurement.

Measurements were carried out in 6 areas—3 areas each on both surfaces. Surfaces were marked A1, B1, C1 on the input side and A2, B2, C2 on the output side. The marking of the measuring areas is shown in Figure 4. For each selected area, 9 profiles 500 μm apart were measured. Surface topography measurements were carried out using PN-EN ISO 4287. Filtration was selected based on ISO 11562; the measurement used a Gaussian filter with a phase correction equal to 0.8 mm.

In the second step, dimensional and shape accuracy were measured using the GOM method on the optical measuring machine Atos ScanBox 6130. The GOM method (Global Optical Measurement) is modern technology that allows precise measurements of product geometry and uses advanced cameras and image analysis software to record and analyze results related to the shape of an object. An optical 3D measuring machine operates on the principle of triple scanning, in which precise stripe patterns are projected onto the surface of an object. Measurement is preceded by camera calibration, during which the measurement system, using a pattern panel, is adjusted to ensure the dimensional consistency of that system. During calibration, the software determines geometric parameters to find the position and orientation of each camera based on the images it records. The beams are recorded by two cameras operating on the stereo camera principle. The paths of the beams from the cameras and the projector are calibrated. It is possible to determine the points of the 3D surface from three different intersections of the beams based on the reflected wave: the camera and the cameras with the visual beam, the camera with the visual beam on the left side and the projector with the projection beam, and the camera with the visual beam on the right side and the projector with the projection beam. Based on the points collected in this way, the software calculates the polygon mesh of the feature surface, as well as the actual values of the control feature plan. These data are compared with the nominal data and presented in a report. The results of the measurements are automatically saved and presented in the form of a color-coded presentation of the deviations according to the assumed scale [42].

**Figure 5:**
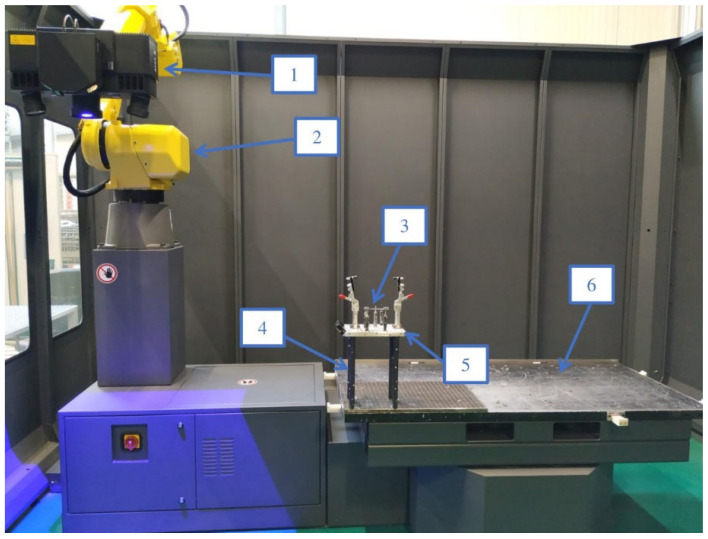
The scheme of measuring dimensional and shape accuracy using the GOM machine: 1—GOM projector, 2—measuring arm, 3—sample, 4—machine posts, 5—universal base, 6—rotary table.

**Figure 6:**
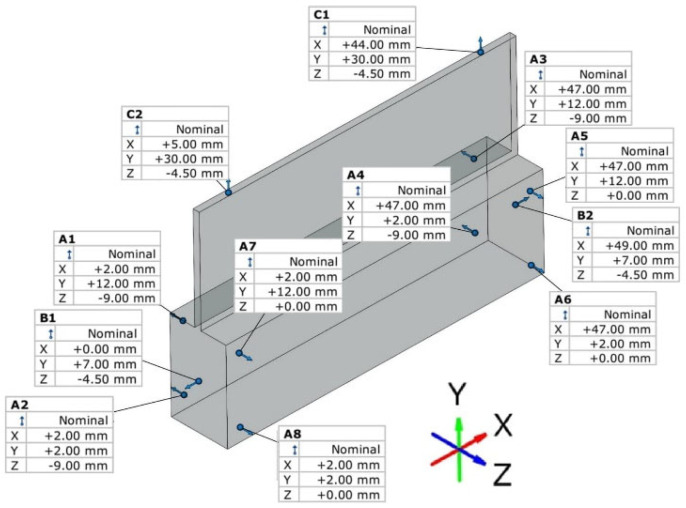
The reference points used during GOM measurement.

The program and series of measurements were carried out using GOM Inspect 2019 software. The samples were mounted on a universal base placed on the posts fixed on the rotary table of the machine. The posts were used to allow easier and free access of the arm with the projector to the measuring point in the space from all points of the sample. The scheme of the described test using the GOM machine is shown in Figure 5. The measurement was carried out in a free state, i.e., none of the samples was fixed. The reference points of the GOM measurement are presented in Figure 6. Fixed points specified B1–B2 were selected to determine basing on the x-axis, points C1–C2 were selected for the y-axis, and points A1–A8 were selected for the z-axis.

**Figure 7:**
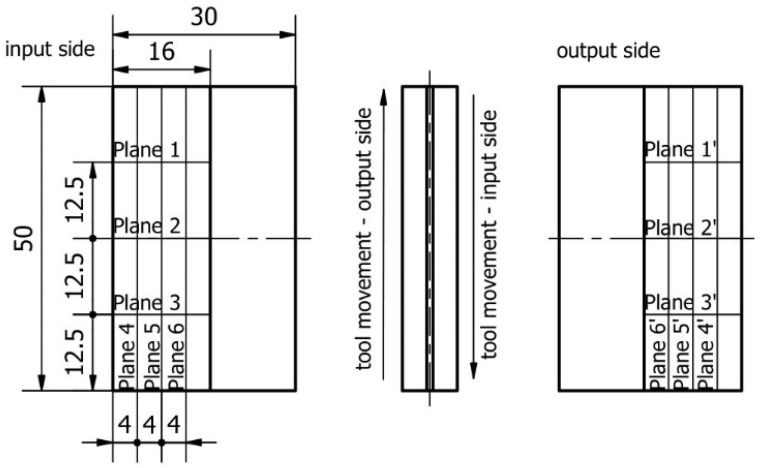
Description of the plane deformation of the sample.

For the samples, cross-sections were defined in 6 parallel and 6 perpendicular directions to the bottom of the sample (3 on each side), determining the deformations on both sides of the machined surface. On the input side the areas are marked from 1 to 6 and on the output side the areas are numbered 1′ to 6′. The method of the base during the measurement and description of the planes are presented in Figure 7.

## 3. Results and Discussion

### 3.1. Surface Topography Analysis

**Figure 8:**
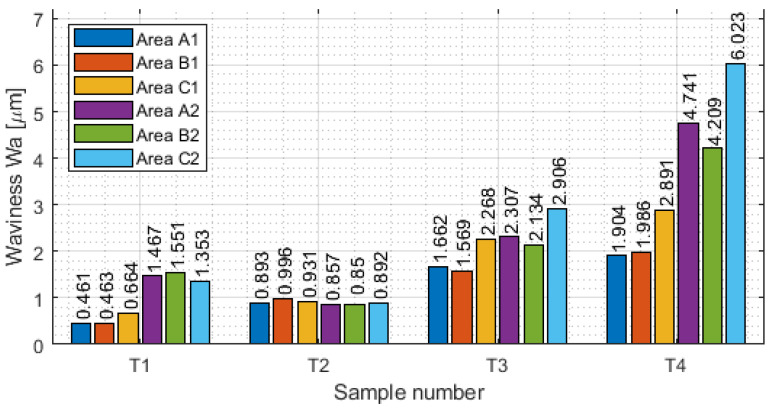
Values of the arithmetic mean waviness W_a_ in areas A1–C1 for the input side and in areas A2–C2 for the output side.

**Figure 9:**
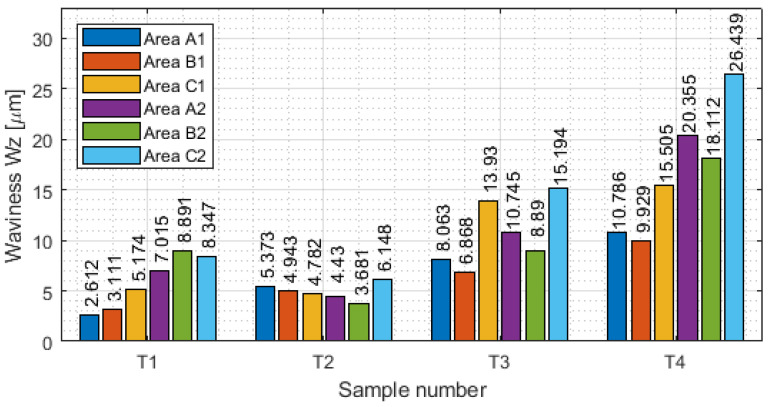
Values of the maximum height of the waviness W_z_ in areas A1–C1 for the input side and areas A2–C2 for the output side.

**Figure 10:**
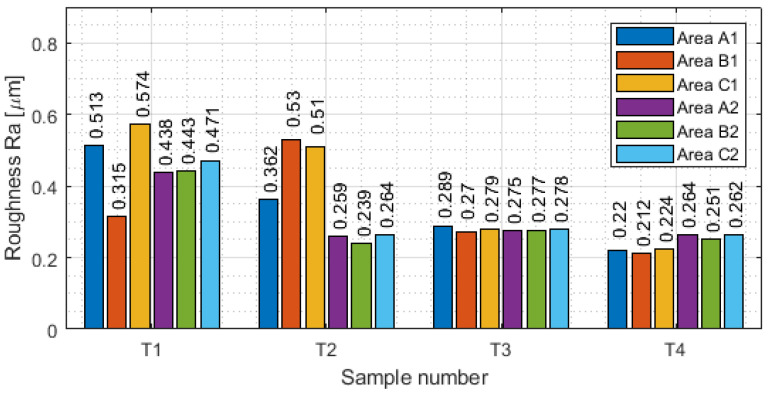
Values of the arithmetic mean deviation R_a_ in areas A1–C1 for the input side and in areas A2–C2 for the output side.

**Figure 11:**
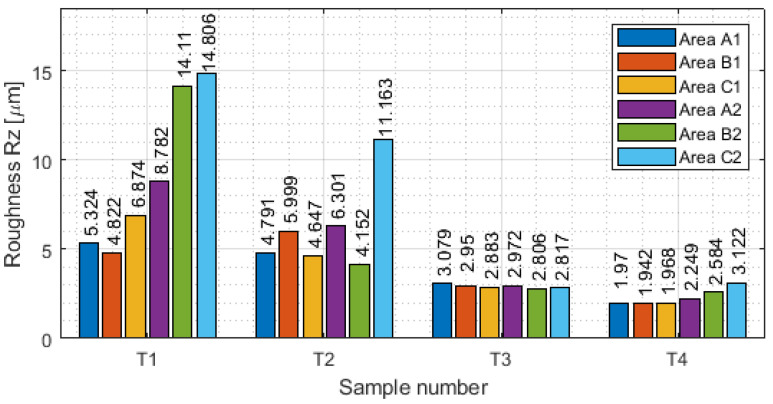
Values of the maximum height R_z_ in areas A1–C1 for the input side and in areas A2–C2 for the output side.

Measurement with a contact allows the generation of the results of the selected basic surface parameters of the tested samples. The values of surface waviness and roughness of the thin-wall surface for each area are shown in Table A1, Table A2, Table A3 and Table A4. Based on the data obtained in these tables, the following were determined as graphical representations of selected parameters (for waviness: W_a_. W_z_ and for roughness R_a_, R_z_) for vertical thin-walled samples in areas A1–C1 for the input side and in areas A2–C2 for the output side. Figure 8 shows the arithmetic mean waviness W_a_, Figure 9 shows the maximum height of the waviness W_z_, Figure 10 shows the arithmetic mean deviation R_a_, and Figure 11 shows the maximum height R_z_.

Comparing the above charts of W_a_ and W_z_ to each other, it is noticeable that the graphs have similar character values. This may indicate that the results are stable and the correct results were obtained. It is observed that there were lower values of waviness for face milling strategies than in cylindrical milling strategies. This was due to less deflection of the thin wall under the influence of the tool. Analyzing individual samples, repeatable values W_a_ and W_z_ are visible for sample T2. Samples T1, T3, and T4 have a dispersion of results, with lower values observed for the input side of the tool into the material. The highest values of waviness were obtained for sample T4. For such a large amount of data, it was difficult to indicate relationships between individual areas. Measurements should be carried out on a larger number of samples to determine a common relationship.

For roughness parameters, it was the opposite tendency than in waviness. The highest values of roughness are visible for sample T1, and the lowest for sample T4. In general, lower waviness was measured for face milling (with greater involvement of radial depth), while higher roughness was obtained. In the face milling strategy, a stepped structure was formed during machining and the boundaries between the passes were perceptible. Roughness values were not repeatable, with no visible correlation between them. This was due to the thick chips, which during processing were pressed into the material and thereby damaged the machined surface. The opposite tendency was observed for cylindrical milling (with greater involvement of the depth of cut) than in face milling. Values of waviness were higher, but values of roughness were observed to be lower. This method obtained a lower roughness because the machined surface was made from a single pass. Higher waviness appeared through the deflection of the tool: it caused a deflection of the sample along the input length. Based on the graphs presented in Figure 10 and Figure 11, for the output side, face milling exhibited higher values of roughness, but for cylindrical milling lower values were observed.

### 3.2. Samples Deformation Analysis

**Figure A1:**
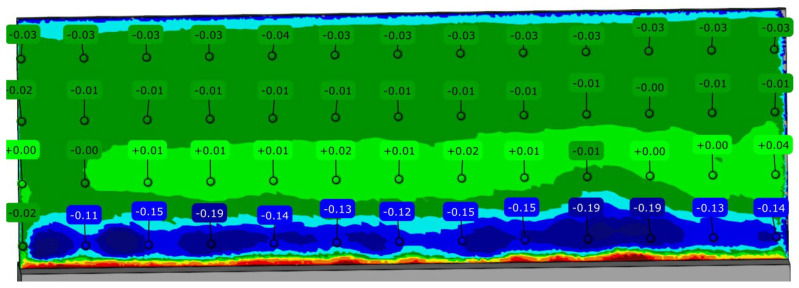
Color map determined using the GOM measurement for sample T1 (input side).

**Figure A2:**
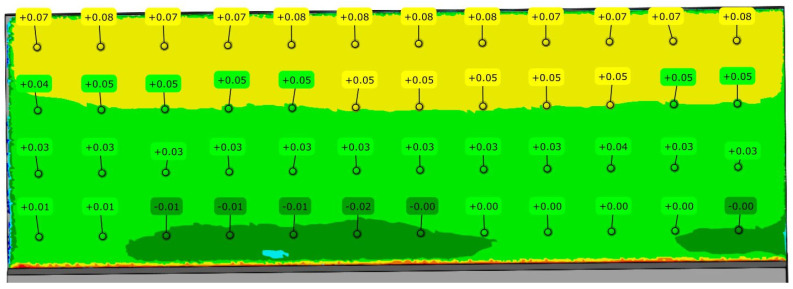
Color map determined using the GOM measurement for sample T1 (output side).

**Figure A3:**
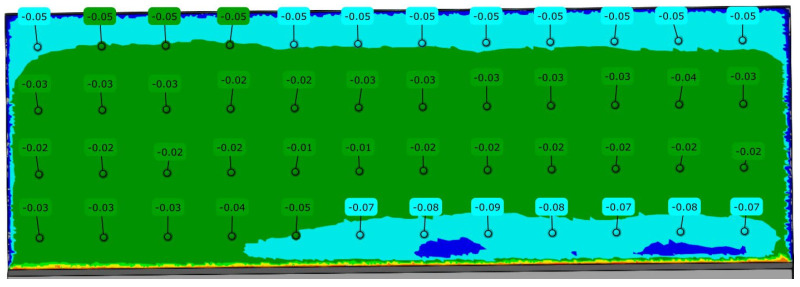
Color map determined using the GOM measurement for sample T2 (input side).

**Figure A4:**
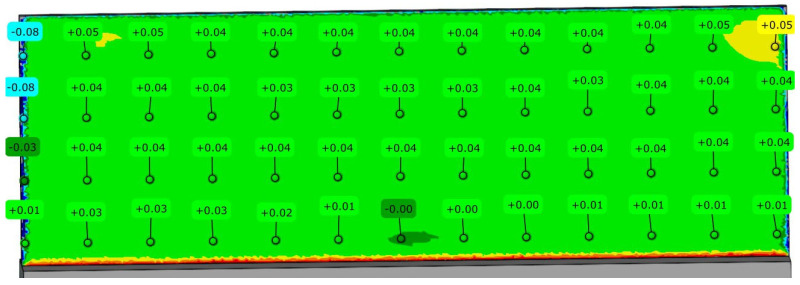
Color map determined using the GOM measurement for sample T2 (output side).

**Figure A5:**
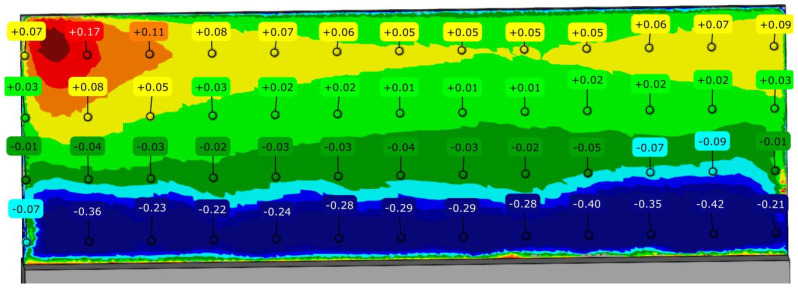
Color map determined using the GOM measurement for sample T3 (input side).

**Figure A6:**
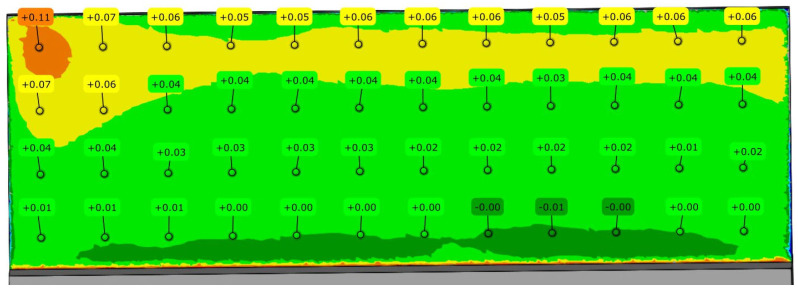
Color map determined using the GOM measurement for sample T3 (output side).

**Figure A7:**
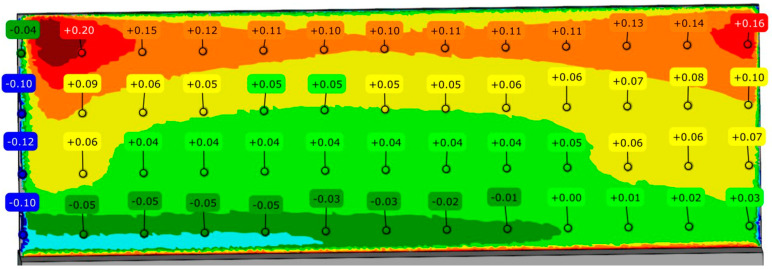
Color map determined using the GOM measurement for sample T4 (input side).

**Figure A8:**
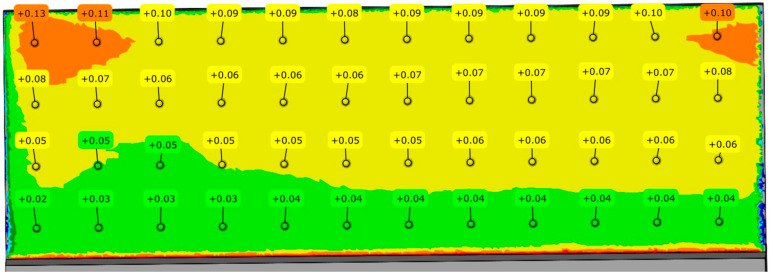
Color map determined using the GOM measurement for sample T4 (output side).

The most common result obtained during optical measurement with GOM is a color map, which presents deviations from the assumed model shape. A minus sign indicates too much material loss, while a plus sign indicates excess material. The color maps for the surface of the tested thin-walled samples are shown in Figure A1, Figure A2, Figure A3, Figure A4, Figure A5, Figure A6, Figure A7 and Figure A8.

**Figure 12:**
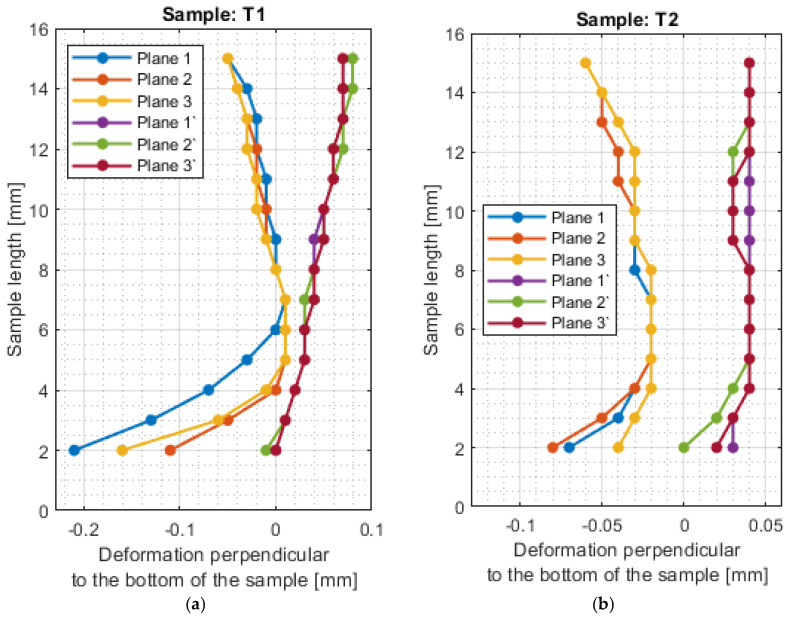
The deformations on the perpendicular direction to the bottom of the sample: (**a**) T1; (**b**) T2.

**Figure 13:**
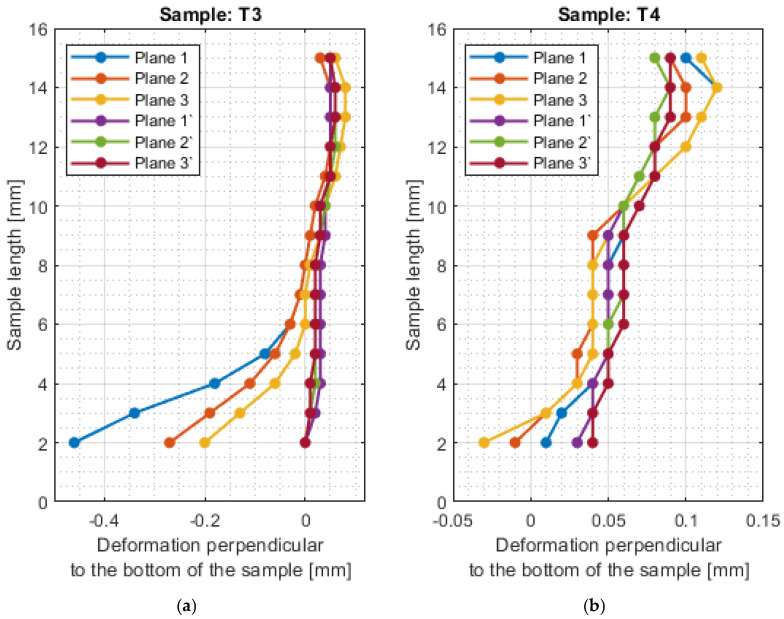
The deformations on the perpendicular direction to the bottom of the sample: (**a**) T3; (**b**) T4.

**Figure 14:**
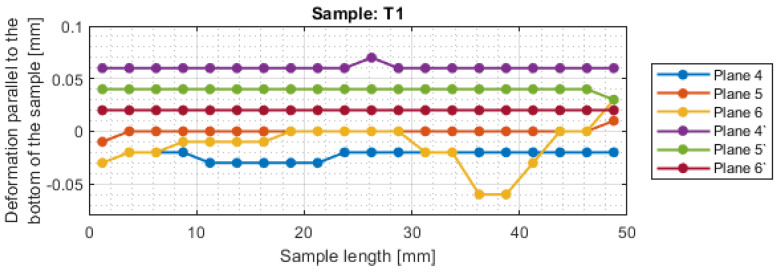
The deformations on the parallel direction to the bottom of the sample T1.

**Figure 15:**
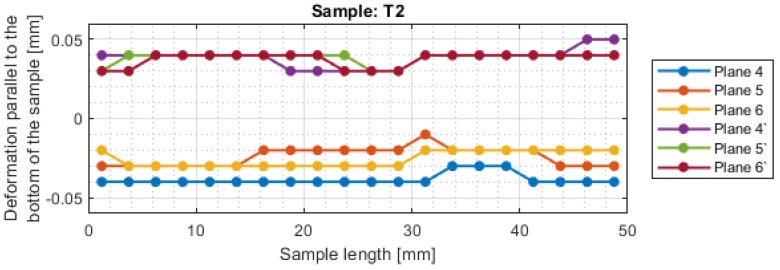
The deformations on the parallel direction to the bottom of the sample T2.

**Figure 16:**
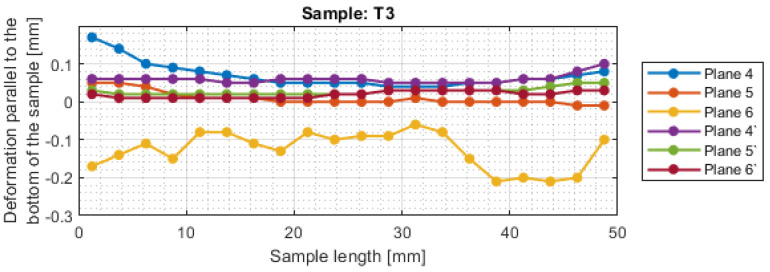
The deformations on the parallel direction to the bottom of the sample T3.

**Figure 17:**
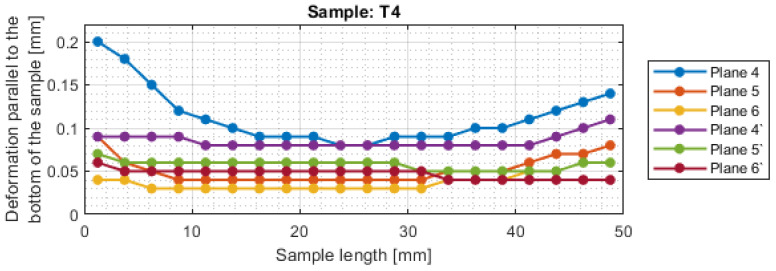
The deformations on the parallel direction to the bottom of the sample T4.

For a more precise determination of deflection in this experiment, the results of the dimensional and shape accuracy test are presented in graphical form as the deformation of the sample in dependence on the length. Diagrams of the sample deformations perpendicular to the bottom of the sample are shown in Figure 12 and Figure 13, and parallel to the bottom of the sample in Figure 14, Figure 15, Figure 16 and Figure 17.

The surfaces obtained after milling with a tool intended for high-performance machining are characterized by a relatively regular shape of the deformation graph in the perpendicular direction to the bottom of the sample. The graphs show small deviations from the nominal value, with lower deviations obtained for the face milling strategy. For sample T2, a sample thickness close to the nominal value was obtained. The deviations shown may be the result of inaccuracies in the adopted method of basing during measurement. The deviations in the perpendicular direction to the sample bottom are lower for the tool for high-performance machining.

The shape deviations are much higher (even four times higher at individual points) for a general-purpose tool. The graph showing the output side of the tool from the material has a relatively regular shape, but on the input side, it is very irregular with significant material losses.

The deviations in the parallel direction to the bottom of the sample are lower for milling with greater involvement of radial depth. It should be borne in mind that the created cross-sections are in the selected zone. Looking at the graphs in the perpendicular direction to the sample bottom, it is presented that the largest deviations occurred at the base of the sample.

A significant material deviation was observed at the beginning of the sample from the tool input side. This is due to the fact that when milling the exit side, the cutter presses on the material, by which it is deformed, and the cutter when entering cut the material from the bent wall. The deformation distribution for the tool indicated for HPM was fairly regular compared with using a tool for general purpose, where a few points with big values of deflection were observed.

Based on the obtained results in the experiment, both for the surface parameters and the deviations, the maximum values of each sample are presented in Table 5. Sample T3 and T4 (for cylindrical milling with greater involvement of depth of cut) had twice lower values of average roughness and twice higher shape deviations compared to face milling. Relatively lower values of both surface topography parameters and deformation were obtained for the tool intended for HPM for both milling strategies.

For sample T2, the value of maximum deviation obtained was lower than the values presented in articles [19,27,28]. It shows that despite the increase in the cross-section of the surface layer, it is possible to obtain a surface with a lower shape deviation. In other cases, the values of maximum deformation were higher. It should be taken into consideration that the specimens were machined with higher parameters than those adopted in these works and that the maximum points in this work appeared mostly at single points. In the studies [19,27,28], deformation was not tested over the entire section, but only at points or over a limited length, so the deviation points may not have been observed.

## 4. Conclusions

In this study, a series of vertical thin-wall samples under controlled process parameters were prepared. Parameters of surface topography (waviness and roughness) and deformations of thin walls were measured using the contact profilometer and optical method (GOM measurement).

It was shown that the optical method could be used for controlling deformations and the general shape of manufactured parts, as well as determining the deflection arrow of a thin-walled sample. When measuring thin-walled samples, it was necessary to perform an accurate scan of the sample to obtain a full point cloud. Based on the obtained point cloud, the results were generated, so if it was not complete then there were errors when making the measurement report.

The experiment also showed that it was possible to use the assumed cutting parameters to make specimens with thin-walled components.

Based on the tests performed, it can be stated that the selection of suitable cutting conditions requires a prior definition of the requirements for the finished product, since the adopted conditions affect the effect differently.

Based on the presented graphs, the following conclusions could be given:

- The experiment showed that waviness, roughness, and deformations take various values in different areas. For detailed quality control, measurement in more zones is required.
- The measurement showed the differences between the input and output sides of the tool into the material. For dimensional and shape accuracy, higher deviations were obtained for the input side. For the waviness and roughness, this could not be determined unequivocally, as it was dependent on the analyzed case.
- GOM measurement is an interesting alternative to current deformation measurement instruments. The resulting point cloud allows inspection of the entire product.
- During the experiment, the following relationship was confirmed:
- The milling strategy had an influence on the selected parameters of surface topography and deformations of the vertical thin-walled parts. On the one hand, the lowest values of waviness and deformations were obtained for cylindrical milling using the tool for HPM, but on the other hand, the smallest roughness was achieved for the same tool using face milling.
- The cutting tool also has an impact on the selected parameters of the thin-walled finished product, but for those selected for the experiment it did not have as much impact as other test conditions. Tests should be carried out for a wider group of tools to determine the appropriate relationships.
- To better understand a process related to thin-walled elements, the authors plan to focus on determining the influence of various materials and plan to measure forces and vibrations during the process to specify a correlation between cutting parameters. Subsequent studies will be carried out on a larger number of samples to provide interrelationships.

## Figures and Tables

**Table 1:** Technical data of tools used in the experiment (own elaboration, based on [37,38]).

Indicator	JSE514100D2C.0Z4-SIRA	JHP770100E2R040.0Z4A-SIRA
Maximum depth of cut	20 mm	20 mm
Coating	SIRON-A	SIRON-A
Cutting diameter	10 mm	10 mm
Number of cutting edges	4	4
Flute helix angle	35°	42°
Lead angle	0°	0°
Overall length	72 mm	75 mm

**Table 2:** The chemical composition of titanium alloy Ti6Al4V.

Element	Ti	Al	V	Fe	O	C
Percentage (%)	balance	5.5-6.75	3.5-4.5	0.4	0.2	max 0.08

**Table 3:** The mechanical properties of titanium alloy Ti6Al4V.

Mechanical Properties	Value	Unit
Yield point R_p0.2_	828	MPa
Tensile strength R_m_	895	MPa
Density	4.43	g/cm^3^
Elongation	10	%

**Table 4:** Depths and description of tools that were used in each case.

Sample	Tool	a_p_ (mm)	a_e_ (mm)
T1	JSE514100D2C.0Z4-SIRA	2	4
T2	JHP770100E2R040.0Z4A-SIRA	2	4
T3	JSE514100D2C.0Z4-SIRA	16	0.5
T4	JHP770100E2R040.0Z4A-SIRA	16	0.5

**Table 5:** The maximum values of selected topography parameters.

Parameter	T1	T2	T3	T4
max W_a_ (μm)	1.551	0.996	2.906	6.023
max W_z_ (μm)	8.891	6.148	15.194	26.439
max R_a_ (μm)	0.574	0.53	0.289	0.264
max R_z_ (μm)	14.806	11.163	3.079	3.122
max deformation (mm)	0.21	0.08	0.46	0.2

## Data Availability

All data are available in the manuscript.

## Appendix A

The values of surface waviness and roughness of vertical thin-walled samples were measured using a contact profilometer for samples T1–T4 (Table A1, Table A2, Table A3 and Table A4). The following tables provide information on waviness and roughness parameters such as: arithmetic mean waviness W_a_, the maximum height of the waviness W_z_, skewness of the waviness profile W_sk_, the the maximum height of peaks of waviness profile W_p_, the the maximum depth of valleys of waviness profile W_v_, the square mean of the waviness deviations W_q_, a kurtosis of the waviness W_ku_, the arithmetic mean deviation R_a_, the maximum height R_z,_ skewness of the roughness profile R_sk_, the maximum height of peaks of roughness profile R_p_, the maximum depth of valleys of roughness profile R_v_, the root mean square roughness R_q_, a kurtosis of the roughness R_ku_.

**Table A1:** Parameters of waviness for input site measured using contact profilometer.

Sample Number	Measuring Area	W_a_ (µm)	W_z_ (µm)	W_sk_	W_p_ (µm)	W_v_ (µm)	W_q_ (µm)	W_ku_
T1	A1	0.461	2.612	1.518	1.185	1.427	0.573	2.580
	B1	0.463	3.111	1.526	1.474	1.637	0.575	2.656
	C1	0.664	5.174	1.769	1.885	3.289	0.872	3.886
T2	A1	0.893	5.373	1.506	3.081	2.292	1.082	2.656
	B1	0.996	4.943	1.45	2.673	2.27	1.232	2.284
	C1	0.931	4.782	1.451	2.516	2.266	1.111	2.373
T3	A1	1.662	8.063	1.332	4.560	3.502	1.946	1.910
	B1	1.569	6.868	1.325	3.617	3.252	1.835	1.874
	C1	2.268	13.93	1.523	9.836	4.094	2.711	2.862
T4	A1	1.904	10.786	1.406	6.137	4.648	2.258	2.226
	B1	1.986	9.929	1.407	5.543	4.477	2.391	2.161
	C1	2.891	15.505	1.374	9.361	6.144	3.413	2.077

**Table A2:** Parameters of waviness for output site measured using contact profilometer.

Sample Number	Measuring Area	W_a_ (µm)	W_z_ (µm)	W_sk_	W_p_ (µm)	W_v_ (µm)	W_q_ (µm)	W_ku_
T1	A2	1.467	7.015	1.370	3.893	3.122	1.724	2.061
	B2	1.551	8.891	1.412	5.112	3.78	1.868	2.188
	C2	1.353	8.347	1.436	5.291	3.056	1.615	2.386
T2	A2	0.857	4.430	1.388	1.765	2.666	0.996	2.186
	B2	0.850	3.681	1.264	1.803	1.878	0.970	1.694
	C2	0.892	6.148	1.441	4.149	2.000	1.057	2.545
T3	A2	2.307	10.745	1.305	6.067	4.678	2.662	1.832
	B2	2.134	8.890	1.283	4.667	4.223	2.447	1.748
	C2	2.906	15.914	1.430	10.594	5.319	3.443	2.366
T4	A2	4.741	20.366	1.282	11.598	8.769	5.415	1.766
	B2	4.209	18.112	1.273	10.707	7.405	4.785	1.738
	C2	6.023	26.439	1.290	14.911	11.528	6.914	1.782

**Table A3:** Parameters of roughness for input site measured using contact profilometer.

Sample Number	Measuring Area	R_a_ (µm)	R_z_ (µm)	R_sk_	R_p_ (µm)	R_v_ (µm)	R_q_ (µm)	R_ku_
T1	A1	0.513	5.324	1.694	2.895	2.428	0.657	3.496
	B1	0.315	4.822	1.875	3.333	1.489	0.413	4.996
	C1	0.574	6.874	1.912	4.341	2.534	0.777	0.462
T2	A1	0.362	4.791	1.845	2.695	2.096	0.472	4.603
	B1	0.53	5.999	1.89	3.489	2.509	0.723	4.359
	C1	0.510	4.647	1.687	2.639	2.009	0.658	3.397
T3	A1	0.289	3.079	1.866	1.507	1.572	0.384	4.317
	B1	0.270	2.950	1.832	1.411	1.539	0.358	4.127
	C1	0.279	2.883	1.81	1.422	1.461	0.368	4.011
T4	A1	0.220	1.970	1.583	1.055	0.915	0.274	2.942
	B1	0.212	1.942	1.593	1.078	0.864	0.265	2.992
	C1	0.224	1.968	1.591	1.061	0.907	0.281	2.964

**Table A4:** Parameters of roughness for output site measured using contact profilometer.

Sample Number	Measuring Area	R_a_ (µm)	R_z_ (µm)	R_sk_	R_p_ (µm)	R_v_ (µm)	R_q_ (µm)	R_ku_
T1	A2	0.438	8.782	2.661	6.257	2.525	0.614	12.315
	B2	0.443	14.11	4.549	8.475	5.635	0.72	35.1
	C2	0.471	14.806	4.619	9.893	4.913	0.792	35.406
T2	A2	0.259	6.301	2.575	4.280	2.021	0.366	12.196
	B2	0.239	4.152	1.990	2.884	1.268	0.320	5.857
	C2	0.264	11.163	7.264	7.775	3.388	0.472	92.499
T3	A2	0.275	2.972	1.834	1.460	1.512	0.363	4.176
	B2	0.277	2.806	1.800	1.446	1.360	0.366	3.946
	C2	0.278	2.817	1.803	1.406	1.411	0.366	3.990
T4	A2	0.264	2.249	1.541	1.140	1.109	0.327	2.747
	B2	0.251	2.584	1.593	1.525	1.059	0.312	3.091
	C2	0.262	3.122	1.626	1.912	1.210	0.330	3.261

## Appendix B

The color maps for the surface of the tested thin-walled samples were obtained during the GOM measurement (Figure A1, Figure A2, Figure A3, Figure A4, Figure A5, Figure A6, Figure A7 and Figure A8).
